# multiMarker: software for modelling and prediction of continuous food intake using multiple biomarkers measurements

**DOI:** 10.1186/s12859-021-04394-z

**Published:** 2021-09-28

**Authors:** Silvia D’Angelo, Isobel Claire Gormley, Aoife E. McNamara, Lorraine Brennan

**Affiliations:** 1 School of Mathematics and Statistics, University College Dublin, Dublin, Ireland; 2 Insight Centre for Data Analytics, University College Dublin, Dublin, Ireland; 3 School of Agriculture and Food Science, Institute of Food and Health, University College Dublin, Dublin, Ireland; 4 Conway Institute, University College Dublin, Dublin, Ireland

**Keywords:** Metabolomics, Biomarker, Intake quantification, R package, Shiny

## Abstract

**Background:**

Metabolomic biomarkers offer potential for objective and reliable food intake assessment, and there is growing interest in using biomarkers in place of or with traditional self-reported approaches. Ongoing research suggests that multiple biomarkers are associated with single foods, offering great sensitivity and specificity. However, currently there is a dearth of methods to model the relationship between multiple biomarkers and single food intake measurements.

**Results:**

Here, we introduce multiMarker, a web-based application based on the homonymous R package, that enables one to infer the relationship between food intake and two or more metabolomic biomarkers. Furthermore, multiMarker allows prediction of food intake from biomarker data alone. multiMarker differs from previous approaches by providing distributions of predicted intakes, directly accounting for uncertainty in food intake quantification. Usage of both the R package and the web application is demonstrated using real data concerning three biomarkers for orange intake. Further, example data is pre-loaded in the web application to enable users to examine multiMarker’s functionality.

**Conclusion:**

The proposed software advance the field of Food Intake Biomarkers providing researchers with a novel tool to perform continuous food intake quantification, and to assess its associated uncertainty, from multiple biomarkers. To facilitate widespread use of the framework, multiMarker has been implemented as an R package and a Shiny web application.

## Background

In the field of Nutrition, self-reported dietary data is associated with a number of well-reported limitations [1]. Recent metabolomic based approaches are investigating biomarkers as more objective measures of food intake which can be used to improve the accuracy of dietary assessment [2]. Recent research effort has focused on identifying new biomarkers of specific foods, with biomarkers emerging for a range of foods including, but not limited to, meat, banana, apple, fish and coffee [3–11]. As the field progresses, it is becoming increasingly evident that multiple biomarkers are needed for a single food to add specificity and sensitivity to the determination of food intake [9]. However, there is a dearth of statistical tools available for modelling multiple biomarkers with a single food. Consequently, the demonstration of the potential utility of multiple biomarkers for food intake determination is hampered. In an effort to address this gap, we have proposed a latent variable approach, multiMarker, to estimate the relationship between food intake and multiple biomarkers, and to subsequently use this relationship to predict intake, together with the associated uncertainty, when only biomarker data are available [12]. The proposed model draws from factor analytic models [13] and mixture of experts models [14], to flexibly model the relationships between biomarkers, discrete food quantities (administered in an intervention study) and latent continuous intakes. To encourage and facilitate usage of the framework by a broad variety of researchers, multiMarker is implemented both in the form of an R package and a Shiny web application. To the best of our knowledge, currently no software allows continuous food intake quantification, and its associated uncertainty, from multiple biomarkers. We believe our software implementations of multiMarker will be useful tools for the research community.

## multiMarker

Here we briefly provide a description of the multiMarker model, to which we will refer when outlining the functionality of the R package and web application in the following Sections.

A factor analytic framework is developed to quantify food intake from multiple biomarkers, obtained from an intervention study, where exact portions of intake are known. For a sample of *n* observations, $i=1,\ldots ,n$, the model expresses the relationship between *P* biomarkers ($\{y_{ip}\}_{p=1}^P$) and latent intake ($z_i$) through a factor analytic model, where the latent variable is the unobserved intake. The latent variable is modelled with a mixture of Gaussian distributions, with components centered around the *D* food quantities administered in an intervention study ($\{x_{d}\}_{d=1}^D$). Further, in order to better refine intake quantification, observation-specific component weights are employed, by embedding the latent intake prior distribution in a mixture of experts context. Thus the multiMarker model is:

$$\begin{aligned} y_{ip}= \alpha _p + \beta _p z_i + \epsilon _{ip}, \quad z_i \thicksim \sum _{d=1}^D \pi _d({\mathbf {y}}_i){\mathcal {N}}_{(0, \infty )} (x_d, \theta _d^2), \end{aligned}$$

where $(\alpha _p,\beta _p)$ are, respectively, biomarker-specific intercept and slope parameters, $\mathbf {\epsilon }_{ip}$ are homoscedastic observation-biomarker-specific error terms, with variance $\sigma _p^2$, and $\theta _d^2$ are scaled component-specific variance parameters. The weights $\pi _d({\mathbf {y}}_i)$ are modelled via an ordinal regression model, with Cauchit link function. The model is developed within a hierarchical Bayesian framework and inference is conducted through Markov chain Monte Carlo (MCMC) algorithms [12]. When only biomarker data are available for $n^*$ observations ($\{y_{jp}^*\}_{p=1}^P$, $j=1,\ldots ,n^*$), after the model parameters have been inferred from the intervention study data, the framework can be employed to perform prediction for the latent intakes $\{z_j^{*}\}_{j=1}^{n^*}$.

## Implementation

The multiMarker R package depends on R ($\ge$ 3.0), and on two further packages: truncnorm (v1.0-8) [15] and ordinalNet (v2.9) [16, 17]. The source code for the R package and a reference manual, containing detailed information on its usage, are available [18].

In the R package, multiMarker and predict.multiMarker are the two main functions. The multiMarker function infers the relationship between multiple biomarkers and food intake; the main arguments of this function are:

- y: a matrix storing *P* biomarker measurements on a set of *n* observations (dimension: $n \times P$);
- quantities: a vector storing the food quantities allocated to each of the *n* observations in the intervention study data (length: *n*);
- niter: the number of MCMC iterations;
- burnIn: the number of MCMC iterations to be discarded prior to computing posterior estimates.

Note that the multiMarker method is independent of both the unit of measure of the *P* biomarkers and of the types of biofluid from which these are measured. The user can freely set the units of measure, and further decide on the biofluids from which to derive the biomarker measurements. Furthermore, the biomarkers can be quantitative or expressed as relative quantities. Model hyperparameters are computed according to the observed data, as described in [12]. However, users can specify different values using additional arguments of the multiMarker function (see [18]). The output of this function is an object of class multiMarker, storing posterior estimates and MCMC chains, for model parameters and latent intakes.

Function predict.multiMarker facilitates prediction of intake values from biomarker data alone; its main arguments are:

- object: an object of class multiMarker;
- y: a matrix storing *P* biomarker measurements on a set of $n^*$ observations (dimension: $n^* \times P$);
- niter, burnIn.

Usage of predict.multiMarker is conditional on the prior estimation of a multiMarker model using data from an intervention study. Moreover, biomarkers considered for prediction should correspond to those of the intervention study, and should be ordered in the same way.

Importantly, in both functions, distributions of parameter estimates and intake predictions are provided, as well as multiple summary statistics: posterior median, posterior standard deviation and $95\%$ credible intervals. This directly provides informative quantification of the uncertainty associated with the different quantities of interest, often lacking from food intake predictions. Examples for the two functions are provided, as well as example code to produce synthetic data, diagnostic plots for the model parameters and plots of the inferred intake distributions.

**Fig. 1:**
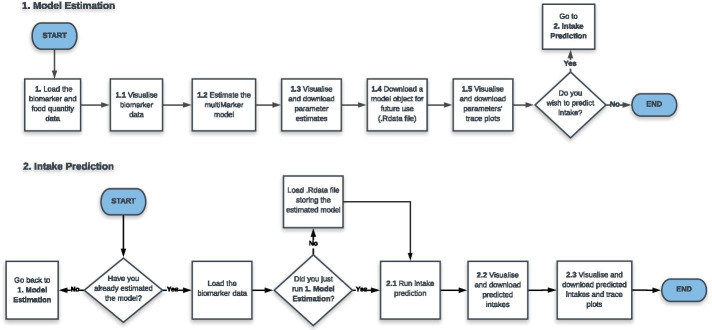
Flowcharts of the “1. Model Estimation” and “2. Intake Prediction” pages of the multiMarker Shiny web application

The multiMarker Shiny web application builds on the R package to provide non-R-expert researchers with easy access to multiMarker. The Shiny application can be accessed at https://adiet.shinyapps.io/multiMarker/. The first two pages of the application, “About” and “Instructions”, contain a brief overview of the web application’s scope and structure. The main pages of the application are “1. Model Estimation” and “2. Intake Prediction”. Figure 1 reports two flowcharts, illustrating the overall structure of the Shiny web application.

In “1. Model Estimation”, users can upload data from an intervention study. Two different data formats are supported: .csv and .txt. Such data should consist of a matrix with *n* rows and $(1 + P)$ columns, with the following structure:

- Column 1: Food quantity values consumed by the participants/observations in a study;
- Columns 2 to $(P+1)$: The *P* biomarker measurements.

Exploratory tools are provided, such as a table containing descriptive summary statistics for the biomarker data, and food quantity-biomarker boxplots (see 1.1 in Fig. 1). Further, intakes’ unit of measure can be specified. The multiMarker model can be run easily using the “Run model estimation” button (see 1.2 in Fig. 1), after having specified the number of MCMC iterations and the desired percentage of iterations for burn-in.

Users are provided with tables containing summary statistics for the estimated intercept ($\alpha _p$), scale coefficient ($\beta _p$) and errors standard deviation ($\sigma _p$) parameters, for the *P* biomarkers. Further, histograms showing the estimated posterior distributions of these parameters can be produced. The estimated model can be downloaded as an R model object, in a .RData file format (see 1.4 in Fig. 1), for future usage. Last, diagnostic trace plots for the parameters can be produced (see 1.5 in Fig. 1).

In “2. Intake Prediction”, users can upload biomarker data. As in “1. Model Estimation”, only .csv and .txt are supported. Uploaded data should consist of a matrix with $n^*$ rows and *P* columns, storing the *P* biomarker measurements. Moreover, if “2. Intake Prediction” is run in a different session than “1. Model Estimation”, users should upload the .RData file storing the previously estimated model. Descriptive summary statistics for novel biomarker data can be found in a table on the left side of the page. Predictions can be carried out using the “Run intake prediction” button (step 2.1 in Fig. 1), after having specified the number of MCMC iterations and the desired percentage of iterations for burn-in. Histograms presenting the posterior predictive intake distributions for each one of the $n^*$ observations can be produced (see 2.2 in Fig. 1). Further, diagnostic trace plots can be accessed, as well as a table with summary statistics for the predicted intakes (see 2.3 in Fig. 1).

All plots produced in “1. Model Estimation” and “2. Intake Prediction” can be downloaded by the users, in a .png format. Last, an example dataset is pre-loaded, which allows users to explore the web application’s functionality.

## Results and discussion

To demonstrate usage of the proposed Shiny web application, we employ results from the analysis of an orange consumption intervention study. Note that analogous output can be retrieved from the R package.

**Fig. 2:**
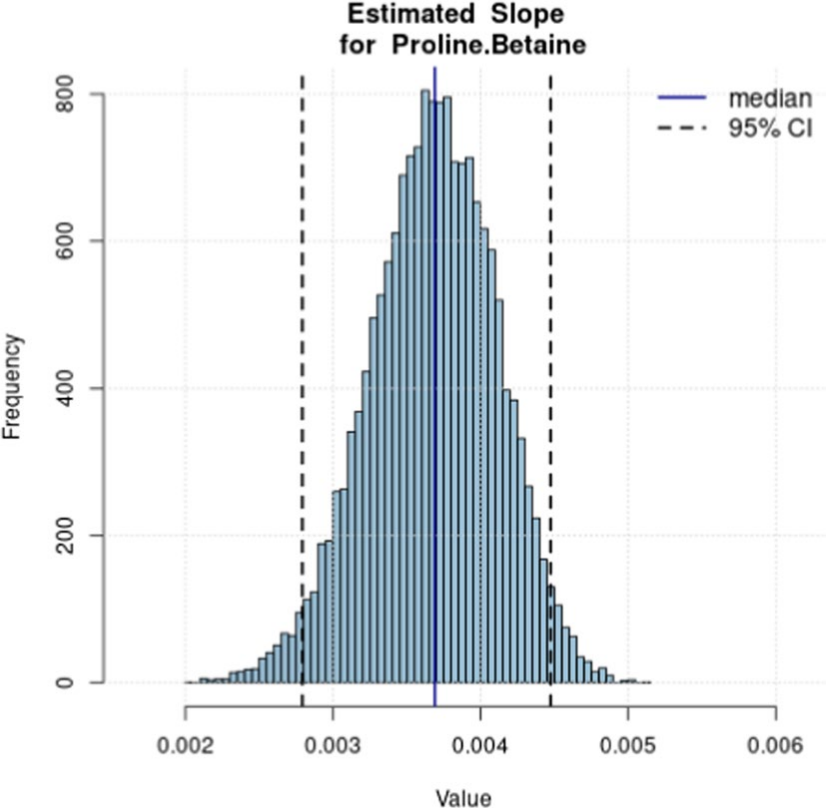
Histogram of the estimated posterior distribution for the slope parameter of the proline betaine biomarker. Estimated posterior median and $95\%$ credible interval are reported

The intervention study data used to estimate the multiMarker model consist of 24, 22 and 24 participants who consumed 80, 160 and 320 grams of orange, respectively. The total number of observations is $n= 24 + 22 + 24 = 70$. Measurements for $P=3$ biomarkers are available: citrate, hippurate, and proline betaine [19]. Full details on the study design and its participants are provided in [10]. While our model does not include study participants’ demographics it is worth noting that future work could examine how to incorporate information such as age and sex, as needed. Intake prediction was performed on a test-set consisting of the same 3 biomarkers for $n^*=3$ observations. The dataset was loaded and visualized in the “1. Model Estimation” page, and the multiMarker model was estimated, setting the number of MCMC iterations and burn in values to 20000 and $15\%$, respectively. The same settings were used in the “2. Intake Prediction” page. Computation time was 2.11 minutes in “1. Model Estimation”, and 25 seconds in “2. Intake Prediction”. All three biomarkers presented similar estimated slope values and associated $95\%$ credible intervals. As an example of plot output of the application, Fig. 2 presents the histogram of the estimated posterior distribution for the slope parameter of the proline betaine biomarker. The estimated posterior median and $95\%$ credible interval are also reported.

**Fig. 3:**
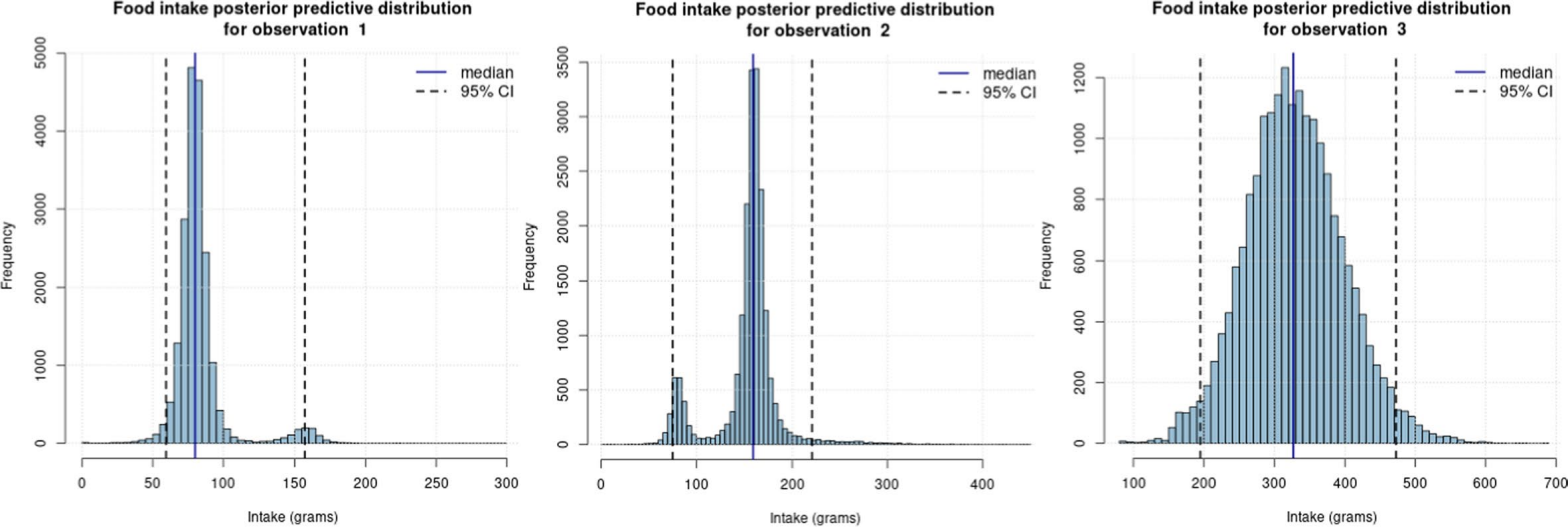
Predicted intake distributions for the three observations in the test data (observations 1, 2 and 3). Median predicted intakes are reported, as well as $95\%$ credible intervals, to account for uncertainty in the food intake quantification

The estimated model from “1. Model Estimation” was then used to predict continuous orange intake for the test data ($n^*=3$). Figure 3 presents an example of output from the “2. Intake Prediction” page, which mimics real world data where only biomarker data are available. The predicted intake distributions for the three observations are displayed, whose actual consumption here is known to be 80 grams (observation 1), 160 grams (observation 2) and 320 grams (observation 3) of orange. The median predicted intakes are in good agreement with the actual intake values: 79.91, 158.72 and 326.43 grams, respectively for observations 1, 2 and 3. Also, note that the estimated predictive distributions of observations 1 and 2’s intake are bi-modal. Here, the model accurately predicts the median intake for this observation, but displays some uncertainty leaning towards either larger (observation 1) or smaller (observation 2) intake values. Credible intervals ($95\%$) are also reported in the output plots, to provide insight on the uncertainty associated with the predicted intakes. The ability to predict continuous intake with an associated uncertainty is an important step forward for the implementation of biomarkers in nutrition research. Further, as biomarkers have limitations, including uncertainty in the estimation of intake will be important for their future use. Last, diagnostic plots (trace plots) are available for all parameters and predicted intakes.

## Conclusion

The multiMarker web application, built on the homonymous R package, allows estimation of the relationship between multiple biomarkers and food intake, and importantly enables users to predict continuous food intake from biomarker data alone. A valuable feature of the work is the estimation of the uncertainty of the food intake predictions, as distributions of both parameter estimates and intake predictions are provided, equipping researchers with more informative quantification of food intake. The web application supports the workflow from data import, analysis and visualisation, thus facilitating the use of multiMarker in the research community. Implementation is available in two different software permitting usage of multiMarker by a broad variety of researchers, from R experts to domain experts. In particular, the multiMarker web application has the potential to facilitate the use of biomarkers for food intake assessment and move the field towards more examples of the utility of such biomarkers. Ultimately, this will pave the way forward for improvement in dietary assessment approaches.

### Availability and requirements

- Project name: multiMarker
- Project home page: https://CRAN.R-project.org/package=multiMarker;
- https://adiet.shinyapps.io/multiMarker/
- https://www.ucdnutrimarkers.com/software
- Operating system: Platform independent
- Programming language: R (v.3)
- Other requirements: truncnorm, ordinalNet (R package); none (Shiny app)
- License: GPL-2, GPL-3
- Any restrictions to use by non-academics: None

## Data Availability

The datasets used and/or analysed during the current study are available from the corresponding author on reasonable request. Example datasets are available on the Shiny Web application: https://adiet.shinyapps.io/multiMarker/.

## Abbreviation

MCMC: Markov Chain Monte Carlo
